# Cell-free DNA release under psychosocial and physical stress conditions

**DOI:** 10.1038/s41398-018-0264-x

**Published:** 2018-10-29

**Authors:** E. M. Hummel, E. Hessas, S. Müller, T. Beiter, M. Fisch, A. Eibl, O. T. Wolf, B. Giebel, P. Platen, R. Kumsta, D. A. Moser

**Affiliations:** 1 Department of Genetic Psychology, Faculty of Psychology, Ruhr-University Bochum, Universitätsstraße 150, 44801 Bochum, Germany; 2 Department of Sports Medicine, Medical Clinic, Eberhard-Karls-University of Tübingen, Otfried-Müller-Straße 10, 72076 Tübingen, Germany; 3 Department of Sports Medicine & Sports Nutrition, Faculty of Sport Science, Ruhr-University Bochum, Universitätsstraße 150, 44801 Bochum, Germany; 4 Department of Cognitive Psychology, Faculty of Psychology, Ruhr-University Bochum, Universitätsstraße 150, 44801 Bochum, Germany; 5 Institute for Transfusion Medicine, University Hospital Essen, University Duisburg‑Essen, Hufelandstraße 55, 45122 Essen, Germany

## Abstract

The understanding of mechanisms linking psychological stress to disease risk depend on reliable stress biomarkers. Circulating cell-free DNA (cfDNA) has emerged as a potential biomarker of cellular stress, aging, inflammatory processes, and cell death. Recent studies indicated that psychosocial stress and physical exercise might also influence its release. We compared the effects of acute psychosocial and physical exercise stress on cfDNA release by exposing 20 young, healthy men to both an acute psychosocial laboratory stressor and an acute physical exercise stressor. Venous blood and saliva samples were collected before and after stress exposure. Cell-free DNA was extracted from plasma and quantified by qPCR. Furthermore, cfDNA fragment length was analyzed and cfDNA methylation patterns were assayed across time. In addition, release of stress hormones and subjective stress responses were measured. Results showed a twofold increase of cfDNA after TSST and fivefold increase after exhaustive treadmill exercise, with an overabundance of shorter cfDNA fragments after physical exhaustion. Interestingly, cell-free mitochondrial DNA showed similar increase after both stress paradigms. Furthermore, cfDNA methylation signatures—used here as a marker for diverse cellular origin—were significantly different post stress tests. While DNA methylation decreased immediately after psychosocial stress, it increased after physical stress, suggesting different cellular sources of active DNA release. In summary, our results suggest stimulus and cell-specific regulation of cfDNA release. Whereas the functional role of stress-associated cfDNA release remains elusive, it might serve as a valuable biomarker in molecular stress research as a part of the psychophysiological stress response.

## Introduction

Increased levels of circulating cell-free DNA (cfDNA) in the bloodstream, either of genomic or mitochondrial origin, are hallmark manifestations of acute systemic inflammatory responses as well as of chronic inflammation. Elevated levels have been reported after trauma, sepsis, stroke, ischemia/reperfusion injury, or myocardial infarction, and in patients suffering from cancer, autoimmune and cardiovascular diseases, as well as metabolic disorders^1–4^. In these conditions, cfDNA has by now been established as a reliable and reproducible biomarker, and quantification of cfDNA levels offers potential as a promising clinical analyte in risk profiling and therapy monitoring in diverse inflammatory settings. Apart from pathological conditions, it has recently been observed that physical exercise acutely triggers an immediate and transient increase in cfDNA^5–9^ but also that exposure to chronic psychosocial stress influences plasma cfDNA levels^10–12^. Both acute bouts of exercise and acute psychosocial stressors provoke immediate neuroendocrine, inflammatory, metabolic, and cardiovascular responses that impact immune homeostasis at multiple levels. It thus appears conceivable that psychosocial stress and mental health conditions should also acutely or chronically impact cfDNA plasma levels. Indeed, recent studies indicate that cfDNA could be a valuable biomarker in the context of psychosocial stress and dysfunction. Increased plasma cfDNA levels were found in animals after exposure to emotional stress as provoked by tail fixation for 18 h^13^. Women undergoing in vitro fertilization treatment who reported high levels of psychosocial distress have been reported to show elevated levels of cfDNA which could be lowered by means of stress reduction intervention^10–12^. Interestingly, recent research has also identified increased numbers of mitochondria following stressful events and during depression, an effect that is thought to be mediated by stress hormones activated through the hypothalamic–pituitary–adrenal (HPA) axis^14,15^. Altered levels of circulating cell-free mitochondrial DNA (cf-mtDNA) in the plasma of suicide attempters and in major depressive disorder have also been described^16–18^. In addition, increased plasma cf-mtDNA levels in suicide attempters were significantly and positively correlated with cortisol levels after dexamethasone suppression, an indicator of hyperactivity of the HPA axis, the organism’s major hormonal stress response system^17^.

To date, cfDNA origin, mechanisms of release, regulation, clearance, and its physiological role are still unclear. Fragment sizes ranging from ~150 bp to larger than 10 kbp have been observed^19,20^. Fragments of 150 bp and multiples of 150 bp are thought to derive from apoptotic processes originating from the endogenous cleavage of chromatin DNA into inter-nucleosomal fragments^21^, whereas larger fragments of ≥10 kbp are thought to derive from necrotic processes. However, active DNA release during disease and after stimulation has also been observed. For instance, cfDNA was found in cell culture supernatant and might act as a potential signaling molecule under distinct conditions^19,22^. Another source of cfDNA are neutrophil extracellular traps (NETs) which represent an ancient and important part of our innate immune defense system^23,24^. NETs are composed of remodeled extracellular DNA fibers that are released by neutrophils in response to pathogenic triggers. Moreover, several white blood cell lineages have by now been reported to be capable of actively releasing DNA, either from nuclear or mitochondrial genomic material^25,26^. Given the associations between increased cfDNA levels and chronic stress as well as markers of stress system dysregulation, we aimed to test whether and to what extent acute psychosocial and physical stress exposure might lead to increased levels of cfDNA and cf-mtDNA in the circulation. We also aimed to test for potential stimulus-specific effects and compared lengths of corresponding cfDNA fragments and their specific methylation pattern as an indicator of different cellular origin after acute psychosocial to physical exercise stress. Lastly, we associated cfDNA levels with release patterns of HPA axis and sympathetic nervous system markers to identify potential release triggers. In addition, emotional responses were measured by means of self-report questionnaires.

## Materials and methods

### Participants

Participants (*n* = 20) were healthy male students of sports science, 18 to 36 years of age (mean = 23.3 ± 3.8 (SD)), with a normal body mass index (mean = 23.4 ± 1.5), no history of or current mental health problems as well as no chronic or acute physical illnesses, and no current intake of medication. All participants gave written informed consent and the study was approved by the local ethics committee (153/2014).

### Procedure

Participants were exposed to both an acute psychosocial laboratory stressor and an acute physical exercise stressor in a randomized order on two different days. Sessions were scheduled for either 9 or 11 am to keep variations in the diurnal cycle of cortisol at a minimum. Induction of psychosocial and exercise stress and the accompanying testing sessions were carried out at least 2 days apart. Half of the participants completed the Trier social stress test (TSST) first, while the other half went through the exercise protocol first, and the order of testing was assigned pseudo-randomly. On arrival, participants filled out a self-report questionnaire on their health status in regard to exercise (Physical Activity Readiness Questionnaire (PAR-Q)^27^) which was reviewed by one of the sports medical physicians on site. A peripheral venous catheter was then inserted on the inside of the participant’s elbow or on their hand by a medical physician (45 min before stress induction), after which participants filled out questionnaires for approximately 25 min, followed by a resting period until the respective stress protocol started. Blood and saliva were sampled 2 min before and 2, 15, 30, and 40 min after cessation of the respective protocol. At four time points (−2, +2, +15, and +30 min), the Social Emotional Response Scale (SERS; Schlotz and Kumsta, unpublished) was completed by the participants. The questionnaire includes 15 questions for the evaluation of arousal (calm, jittery, tense, intense, relaxed, content), self-directed emotions (guilty, ashamed, blameworthy, angry at self, dissatisfied with self), and anxiety (fearful, worried), rated on a scale ranging from 1 = not at all to 4 = a lot.

### Induction of psychosocial stress

Psychosocial stress was induced by means of the TSST as described elsewhere^28^. In brief, the TSST consists of a preparation period, a free speech, and an unanticipated math task performed in front of a panel of judges and a camera. The TSST is a very well-validated and widely used standardized acute laboratory stressor. As such, it has repeatedly been shown that it reliably activates the HPA axis and in turn leads to significant elevations of the stress hormone cortisol^29^ which has been attributed in large part to the elements of uncontrollability and social-evaluative threat immanent to the situation^30^.

### Induction of physical exercise stress

An exhaustive treadmill exercise with a 15% incline was carried out in order to keep the duration to 10 to 15 min, as similar as possible to that of the TSST. It started out with a 5 min walking period at 1 m/s, after which a stepwise increase of speed by 0.2 m/s was introduced every 30 s until the participant reached subjective exhaustion, at which point the treadmill was stopped and the stress induction was thus concluded.

### Plasma preparation and cfDNA extraction

In a recent overview, El Messaoudi et al.^31^ compared pre-analytical factors influencing cfDNA quality from the moment of blood drawing to storage of extracted cfDNA. According to their recommendations, 5 ml of whole blood was collected in EDTA-collection tubes (EDTA Monovettes, Sarstedt, Germany) at each time point and was immediately centrifuged at 1600 × *g* and 4 °C for 10 min. Plasma was transferred to a fresh tube followed by a second 10 min of centrifugation at 16,000 × *g* and 4 °C. Finally, plasma was passed through a 0.8 µm filter and aliquots were stored at −80 °C until further analysis.

The QIAamp Circulating Nucleic Acid Kit (Qiagen, Hilden, Germany), which is considered the gold standard for cfDNA extraction^32,33^, was used to extract cfDNA from 0.9 ml plasma according to the manual provided with the kit. Cell-free DNA was eluted in a final volume of 100 µl H_2_O.

### Spike-in preparation

To control for constant extraction efficiencies, all plasma samples were spiked with plasmid DNA at defined copy numbers as described below. This spike-in control DNA was generated from a 3493 bp pMK-RQ cloning vector, carrying a DNA fragment of the pigeon (*Columba livia*), activity regulated cytoskeleton associated protein (Arc; XM_005510918.1; kindly provided by Dr. Rena Klose). Plasmid copy numbers were calculated using the DNA copy number and dilution calculator (www.thermofisher.com). Subsequently, 2 µg plasmid DNA was digested using *Tsp45I* (NEB, Frankfurt am Main, Germany), generating fragments of 103 bp, 306 bp, 663 bp, and 2,421 bp from the circular plasmid. Reaction was heat-inactivated at 65 °C for 15 min, diluted to 1 × 10^6^ copies/µl, aliquotted, and frozen at −20 °C. Completeness of digestion was controlled using an Agilent DNA 1000 kit on an Agilent 2100 Bioanalyzer. Each plasma sample was spiked with 400,000 copies of fragmented plasmid and eluted in 100 µl H_2_O, resulting in 4000 plasmid copies/µl. Percentage efficiency of extraction was controlled by quantitative polymerase chain reaction (qPCR) targeting plasmid fragments compared to its specific standard curves, ranging from 2.5 × 10^2^ to 2.5 × 10^5^ copies.

### Quantitative PCR

The qPCRs for the assessment of cfDNA, cf-mtDNA, and spike-in controls were carried out on a CFX384 Touch™ Real-Time PCR Detection System (BioRad, Hercules, USA). Primers for cfDNA, cf-mtDNA, and spike-in control were designed to produce target-specific amplicons of 70–110 bp (see supplementary information 1). The limits of detection (LOD) and the limits of quantification (LOQ) were determined as described elsewhere^34^. As pre-tests revealed that cfDNA extraction was highly efficient for different fragment sizes (supplementary information 2), and in order to save cfDNA material for other applications, only the 103 bp ARC fragments were used as a reporter of cfDNA extraction efficiency. CfDNA displays a reasonably good representation of the whole genome with a relative amount of all genomic features in cfDNA of approximately 1^35^. Therefore, we used an in-house BDNF assay (brain-derived neurotrophic factor) that is usually used for high resolution melting (HRM) genotyping of rs6265A/G. This in-house qPCR assay combines high affinity with high linearity, highly satisfying LOD/LOQ and no primers detectable by melting curve analysis for the no-template controls (NTCs). The qPCR reaction for the quantification of cfDNA, cf-mtDNA, and spike-in control contained 0.2 µM primers (see supplementary information 1) and 5 µl iTaq Universal SYBR Green Supermix (BioRad, Hercules, USA) in a total volume of 10 µl. The standard amplification protocol included an initial denaturation step for 3 min at 95 °C, followed by 40 cycles of melting at 95 °C for 5 s, annealing and extension at 60 °C for 20 s, and a melting curve analysis. Triplicate qPCR assays were performed for each point of a cfDNA- and cf-mtDNA-specific standard curve (generated from artificial human DNA and mtDNA gene fragments; MWG Eurofins, Ebersberg) compared to the unknown study samples. All experiments included NTCs with the addition of diluent without cfDNA extract or *Arc* plasmid, and plate normalization was achieved using an interplate calibrator.

### Cell-free DNA fragment analysis

To test for potential differences in cfDNA fragment length after psychosocial vs. physical stress tests, cfDNA samples were analyzed on a Fragment Analyzer (Advanced Analytical, Heidelberg, Germany) using the DNF-488 HS Genomic DNA Kit (Prerun: 6.0 kV, 30 s; Sample injection: 9 kV, 30 s; Separation: 6.0 kV, 50 min). This test enables the analysis of minute amounts of DNA (50 pg/µl) for their respective size in the range of 50 bp to 40 kbp. Samples were analyzed in the Genomics and Transcriptomics Laboratory of the University of Düsseldorf (BMFZ/GTL Professor Karl Köhrer).

### DNA methylation analysis

In order to test for potential differences in cellular origin of circulating cfDNA after stimulation, DNA methylation patterns of the *homeobox A5 gene* (*HOXA5*) over time were compared between conditions. *HOXA5* was chosen for methylation analysis because of its highly cell type-specific DNA methylation patterns, showing hypermethylation in muscle cells, hypomethylation in brain cells deriving from the hippocampus and cultured neuronal cell lines, and differential methylation in blood cells (http://www.roadmapepigenomics.org/; http://www.blueprint-epigenome.eu/). cfDNA (20 µl) was subjected to bisulfite conversion (EZ-96 DNA Methylation-Lightning™ Kit, Zymo Research) and eluted in 10 µl H_2_O. Using primers as indicated in supplementary information 1, a 173 bp *HOXA5* gene fragment located in the CpG island of exon1 (chr7:27,182,652–27,182,824) was amplified on a T100™ Thermal Cycler (BioRad, Hercules, USA). For amplification, 2.5 µl bisulfite-modified DNA, 0.2 µM primers (see supplementary information 1), and 15 µl GoTaq® G2 Hot Start Green Master Mix (Promega GmbH, Mannheim, Germany) were mixed in a total volume of 30 µl. The amplification included an initial denaturation step of 2 min at 95 °C, followed by 50 cycles of melting at 95 °C for 30 s, annealing at 56 °C for 45 s, extension at 74 °C for 45 s, and a final extension step at 74 °C for 10 min. PCR products were purified using 1.5 µl of a sepharose beads suspension (GE Healthcare, UK). Quantitative methylation analysis was performed on a Pyromark Q24 Advanced system (Qiagen, Hilden, Germany) comparing individual DNA signatures over time for the different stress paradigms tested. To ensure that the assay was not biased toward methylated or non-methylated DNA, it was validated using a standard curve of DNA with known methylation (100%; 75%; 50%; 25%; 0%). Methylation standards were created using DNA methyltransferase (M.SssI; NEB, Frankfurt am Main, Germany), and non-methylated standards were generated using the REPLI-g Mini Kit (Qiagen, Hilden, Germany) according to the manuals.

### Hormonal analysis

Plasma and salivary cortisol were analyzed on a Synergy2 plate reader (Biotek, USA) using commercial enzyme-linked immunosorbent assays (ELISAs; cortisol and free cortisol in saliva; Demeditec, Germany) according to the manufacturer’s instructions. Intra- and interassay variability were less than 7% and 8.5% for plasma cortisol and less than 5% and 7% for salivary cortisol, respectively. Salivary alpha-amylase (sAA) activity^35^ was measured as described elsewhere^36^, and showed an intra- and interassay variability of less than 4% and 5%, respectively. Catecholamines, in this case adrenaline (A) and noradrenaline (NA), were commercially high-performance liquid chromatography (HPLC)-measured by LSM (Labor für Stressmonitoring, Göttingen, Germany). To estimate plasma catecholamine concentrations, a solvent extraction system for the selective and quantitative isolation of A and NA from a sample matrix was used. The clean-up procedure was adopted from Smedes et al.^37^ and slightly modified for the use of lower sample volumes.

### Statistical analysis

Statistical analysis was performed with the statistical program SPSS (version 20, SPSS Statistics/IBM Corp., Chicago IL, USA). The data were analyzed with repeated measures analysis of variance (ANOVA; with Greenhouse–Geisser correction for violation of the assumption of sphericity). The effect sizes were reported as eta (ƞ2). In case of significant effects, Fisher's least significant difference (LSD) test was carried out to check for group differences. Some of the parameters were not normally distributed and therefore transformed. A natural logarithm (ln) transformation was applied to cfDNA, cf-mtDNA, alpha-amylase, adrenaline, noradrenaline, plasma, and salivary cortisol data.

## Results

### cfDNA

**Fig. 1:**
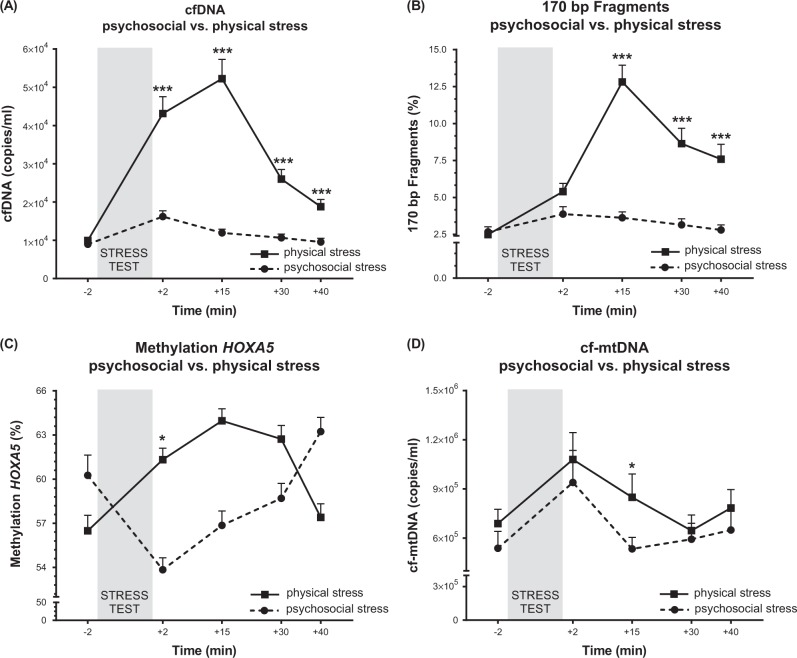
cfDNA (concentration, methylation, fragment length) and cf-mtDNA concentrations before and after psychosocial and physical stress are shown. **a** Changes in cfDNA concentrations (copies/ml plasma) before and after psychosocial and physical stress. Immediately after the TSST, doubling of cfDNA concentration was observed, whereas it increased fivefold after physical stress, peaking 15 min after the cessation of exercise. **b** Changes in the percentage proportion of the 170 bp cfDNA fragments of purified cfDNA. While minimal increases of the 170 bp fragments could be observed after the TSST, a fivefold percentage increase 15 min after physical exercise was observed. **c** Percentage cfDNA methylation of a HOXA5 fragment is shown as the average of 9 CpG sites. DNA methylation increased by 7.5% after physical stress, while it significantly decreased by 6.5% after the TSST. **d** An almost twofold increase of cf-mtDNA directly after both stress conditions is shown. Post-hoc test showed significant difference between the stress paradigms at time point +15 min. Values are reported as means ± SEM. The data were analyzed with repeated measures ANOVAs. In case of significant effects, post-hoc test was carried out to check for group differences (*p ≤ 0.05, ***p ≤ 0.001)

Quantification of circulating cfDNA revealed significantly increased levels after both stress paradigms (main effect time: *F*_(3.22, 122.49) _= 101.44, *p* < 0.001, η^2^ = 0.727). Immediately after cessation of psychosocial stress, cfDNA significantly increased from ~8900 copies of cfDNA per milliliter plasma (copies/ml) to more than 16,200 copies/ml. Following the physical stress condition, a fivefold increase in circulating cfDNA with peak levels at 15 min after maximal physical strain was observed (see Fig. 1a). Magnitude of cfDNA release and response dynamics, i.e., differences in the timing of peak levels, differed significantly between conditions (main effect condition: *F*_(1, 38) _= 51.98, *p* < 0.001, η^2^ = 0.578; time × condition interaction effect: *F*_(3.22, 22.49)_ = 30.42, *p* < 0.001, η^2^ = 0.445). Post-hoc test showed significant difference between conditions at all measured time points after the stress tests (*p* < 0.001). CfDNA rapidly decreased after reaching peak levels and returned close to baseline levels within the time frame tested, which is consistent with the cfDNA half-life of ~15 min^38^.

### Fragment analysis

Before stress exposure, DNA fragment analysis showed that 6.6% of cfDNA fragments consisted of fragments smaller than 300 bp, most of about 170 bp in size. Thus, 92.6% of the cfDNA fragments had a size between 300 bp and 1.500 bp, and less than 1% were longer than 1500 bp, with a maximum fragment length of 40 kbp, and were not further analyzed (see supplementary information 3 for fragment abundance over time). As shown in Fig. 1b, the ~170 bp fragments increased significantly after both stress conditions. Repeated measures ANOVA showed a significant effect of time (*F*_(3.04, 115.65)_ = 36.45, *p* ≤ 0.001, η^2^ = 0.490), an effect of condition (*F*_(1, 38)_ = 35.84, *p* ≤ 0.001, η^2^ = 0.485), and a time by condition interaction (*F*_(3.04, 115.65)_ = 33.77, *p* ≤ 0.001, η^2^ = 0.471) for the ~170 bp fragments. Post-hoc test showed significant difference between conditions at time points +15 min, +30 min, and +40 min after stress test (*p* < 0.001). Analysis for the fragments between 300 and 1500 bp showed no significant effects.

### Epigenetic analysis of *HOXA5*-specific cfDNA fragments

In order to test for differences in cellular origin of cfDNA between conditions, we used *HOXA5* as a reporter gene, as it displays significant tissue-specific DNA methylation. As shown in Fig. 1c, changes in DNA methylation patterns over time followed opposite directions (time × condition interaction effect: *F*_(4, 120) _= 7.63, *p* < 0.001, η^2^ = 0.203; effect of time: *F*_(4, 120) _= 1.68, *p* = 0.159, η^2^ = 0.053; effect of condition: *F*_(1, 30) _= 0.006, *p* = 0.94, η^2^ = 0.000). Post-hoc test showed significant difference between conditions directly after the stress tests (*p* = 0.033). After TSST, DNA methylation decreased from baseline by 6.5% before it increased again, whereas it increased by 7.5% after physical exercise.

### Cf-mtDNA

Immediately after both stress paradigms, significant elevations of cf-mtDNA were observed (effect of time: *F*_(3.14, 119.18) _= 4.82, *p* = 0.003, η^2^ = 0.113; time × condition interaction effect: *F*_(3.14, 119.18) _= 1.60, *p* = 0.192, η^2^ = 0.040; effect of condition: *F*_(1, 38) _= 1.80, *p* = 0.188, η^2^ = 0.045), with a 1.7-fold increase after TSST and 1.6-fold increase after physical stress. After both stress situations cf-mtDNA decreased rapidly back to baseline levels within 30 min, as shown in Fig. 1d. A significant difference between conditions at time point +15 min was found in the post-hoc test (*p* = 0.035).

### Hormonal activation

**Fig. 2:**
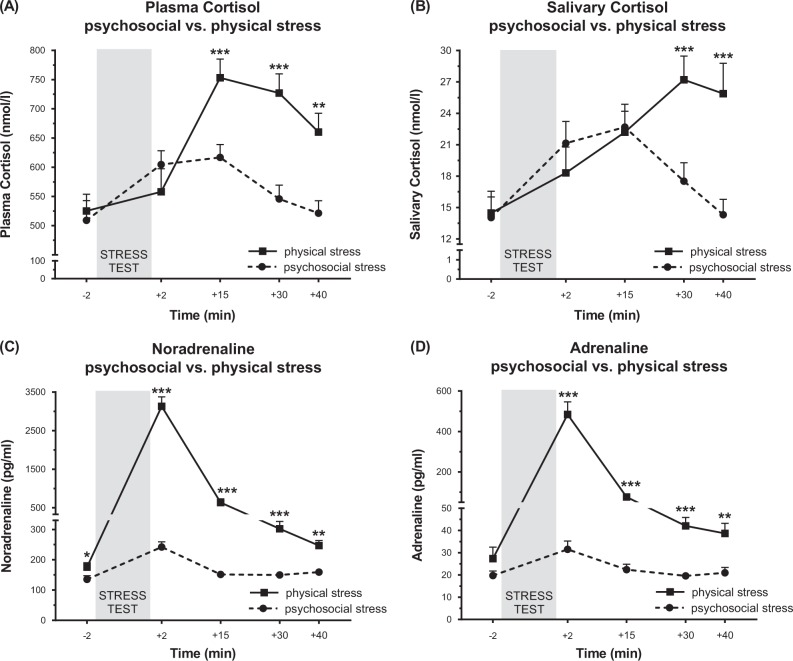
Hormone levels before and after psychosocial and physical stress conditions. **a**–**d** Progression of stress hormones in plasma and saliva before and after psychosocial and physical stress are shown. **a** Plasma cortisol is plotted in nmol/l over time. Both stress conditions led to an increase in plasma cortisol with the highest values measured 15 min after cessation of psychosocial and physical stress. Plasma cortisol increased by 107 nmol/l after psychosocial stress and by more than 210 nmol/l after physical exhaustion (main effect time: *F*_(1.96, 74.48) _= 25.26, *p* < 0.001, η^2^ = 0.399; main effect condition: *F*_(1, 38) _= 4.57, *p* = 0.039, η^2^ = 0.107; time × condition interaction effect: *F*_(1.96, 74.48) _= 13.50, *p* < 0.001, η^2^ = 0.262). **b** Increased salivary cortisol (in nmol/l) in psychosocial and physical stress conditions. Cortisol levels after psychosocial stress peaked after 15 min, whereas they increased until 30 min after physical stress (main effect time: *F*_(1.83, 69.58) _= 17.47, *p* < 0.001, η^2^ = 0.315; main effect condition: *F*_(1, 38) _= 2.20, *p* = 0.146, η^2^ = 0.055; time × condition interaction effect: *F*_(1.83, 69.58) _= 11.10, *p* < 0.001, η^2^ = 0.226). Psychosocial and physical stress led to an increase of noradrenaline (**c**) and adrenaline (**d**). Immediately after psychosocial stress, the concentration of noradrenaline and adrenaline doubled before dropping back to baseline levels within 15 min. Physical stress led to an 18-fold increase of noradrenaline (**c**) and adrenaline (**d**) (noradrenaline: main effect time: *F*_(2.21, 83.89) _= 226.57, *p* < 0.001, η^2^ = 0.856; main effect condition: *F*_(1, 38) _= 152.10, *p* < 0.001, η^2^ = 0.800; time × condition interaction effect: *F*_(2.21, 83.89) _= 104.94, *p* < 0.001, η^2^ = 0.734; adrenaline: main effect time: *F*_(2.21, 73.04) = _103.06, *p* < 0.001, η^2^ = 0.757; main effect condition: *F*_(1, 33) _= 48.54, *p* < 0.001, η^2^ = 0.595; time × condition interaction effect: *F*_(2.21, 73.04) _= 55.80, *p* < 0.001, η^2^ = 0.628). Values are reported as means ± SEM. The data were analyzed with repeated measures ANOVAs. In case of significant effects, post-hoc test was carried out to check for group differences (*p ≤ 0.05, **p ≤ 0.01 ***p ≤ 0.001)

Significant increases were observed for all hormones (effect of time: all *F* > 13.19, all *p* < 0.001, η^2^ > 0.258; see Fig. 2 for response curves). Furthermore, we observed differences in magnitude and reaction curve patterns between conditions for all measures, with larger increases following physical exercise (time × condition interaction effect: all *F* > 11.10, all *p* < 0.001, all η^2^ > 0.226; main effect condition: all *F* > 2.20, all *p* < 0.146, all η^2^ > 0.055), except for sAA (see supplementary information 4).

### Emotional response

Analysis of psychosocial responses showed an increase in tense arousal following both stress situations (effect of time: *F*_(2.20, 81.34) _= 66.85, *p* < 0.001, η^2^ = 0.644; effect of condition: *F*_(1, 37) = _5.81, *p* = 0.021, η^2^ = 0.136; time × condition interaction effect: *F*_(2.20, 81.34)_ = 2.72, *p* = 0.067, η^2^ = 0.069). Post-hoc test showed significant difference between conditions at time points +2 min (*p* = 0.013) and +15 min (*p* = 0.008). Self-directed emotions and anxiety, however, increased only during psychosocial stress conditions, whereas these emotions remained unchanged after physical stress (self-directed emotions: effect of time: *F*_(1.84, 67.95)_ = 13.38, *p* < 0.001, η^2^ = 0.266; effect of condition: *F*_(1, 37)_ = 6.75, *p* = 0.013, η^2^ = 0.154; time × condition interaction effect: *F*_(1.84, 67.95)_ = 6.76, *p* = 0.003, η^2^ = 0.154; anxiety: effect of time: *F*_(2.10, 77.70)_ = 4.11, *p* = 0.019, η^2^ = 0.100; effect of condition: *F*_(1, 37)_ = 9.21 *p* = 0.004, η^2^ = 0.199; time × condition interaction effect: *F*_(2.10, 77.70)_ = 2.87, *p* = 0.060, η^2^ = 0.072; see supplementary information 5). Post-hoc test showed significant differences between conditions for self-directed emotions directly after stress condition (*p* < 0.001) and for anxiety at time points −2 min (*p* = 0.004), +2 min (*p* = 0.003), and +15 min (*p* = 0.018).

### Correlation between hormones and cfDNA

**Fig. 3:**
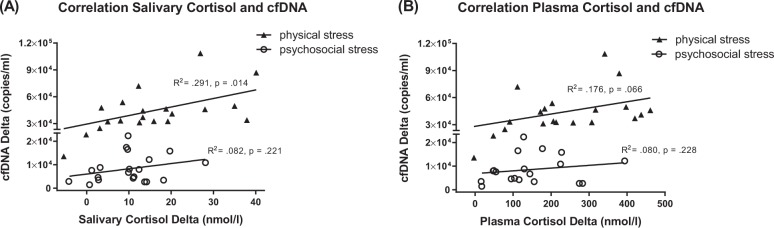
**a** A significant positive correlation between salivary cortisol increases and cfDNA increases (peak minus baseline) was observed in the physical stress condition. The association between increases in salivary cortisol and cfDNA levels after the TSST followed the same direction but was not statistically significant. **b** A trend toward significance for a positive correlation between plasma cortisol and cfDNA increase after physical stress and a similar, non-significant relationship for the TSST was observed

As illustrated in Fig. 3a, a significant positive correlation was observed between salivary cortisol increase and cfDNA increase in the physical stress condition (*r* = 0.539, *R*^2^ = 0.291, *p* = 0.014), with a similar relationship between plasma cortisol increase and cfDNA increase at trend level (Fig. 3b: *r* = 0.419, *R*^2^ = 0.176, *p* = 0.066). Correlations with indicators of SNS activity and cfDNA did not show significant effects, although there was a positive association between cfDNA increase and adrenaline increase in the physical stress condition (*r* = 0.381, *R*^2^ = 0.145, *p* = 0.097). There were no significant correlations between any of the hormones and cfDNA in the TSST condition.

### Correlation between hormones and cf-mtDNA

No correlation was observed between increases in cf-mtDNA and cortisol or catecholamines in either condition. However, when considering individual measuring points, a correlation between cf-mtDNA and adrenaline 15 min after physical stress could be found (*r* = 0.451, *R*^2^ = 0.203, *p* = 0.046).

## Discussion

Stress-related mental and physical disorders have seen a continuous rise in recent years and entail a tremendous societal socio-economic burden. The understanding of the mechanisms linking psychosocial stress to disease risk depend on reliable stress biomarkers. Increased levels of cell-free DNA, an emerging biomarker for a range of pathological conditions, have recently been associated with the experience of chronic stress. Here, we show for the first time that exposure to acute psychosocial stress leads to immediate and transient increases in cfDNA levels.

In contrast to cfDNA, the magnitude of cf-mtDNA release was similar between psychosocial and physical provocation. Mitochondria express glucocorticoid receptors^39^ providing for a potential functional link between stress exposure and cf-mtDNA biology. Recently, elevated cf-mtDNA levels in plasma were found in individuals who had attempted suicide, and these blood cf-mtDNA levels correlated with high post-dexamethasone cortisol levels^16^, supporting an association between HPA axis activity and mitochondrial function. In our study of healthy young men, however, no association could be observed between cortisol and cf-mtDNA release in either condition. Cf-mtDNA levels have also been assessed in patients diagnosed with mental disorders, with mixed results. In patients with major depressive disorder, both elevated^17,18^ and decreased levels^16^ have been reported. Furthermore, Jiang et al.^40^ recently reported increased mtDNA levels in schizophrenic patients, whereas they found it unaffected in patients with mood disorders. These inconsistent results might in part be due to technical confounders in the quantification of cf-mtDNA fragments, including extraction methods and amplicon size. In contrast to extracellular genomic DNA, which is at least partially protected from enzymatic degradation by nucleosomal packaging, mtDNA when freely exposed to blood plasma is highly vulnerable to complete degradation by serum DNase I. Cf-mtDNA will thus be rapidly degraded to fragment sizes below the detection limits of PCR-based methods, as recently shown in plasma from sepsis patients^41^. Exercise-triggered transient rise in serum DNase I activity will further speed up this process under physical stress conditions^7^. Thus, it cannot be excluded that we and others underestimated the true release kinetics of mtDNA under various settings^6,42,43^, which might also explain the lack of correlation between cortisol and catecholamines and cf-mtDNA and highlight the importance of standard plasma DNA detection procedures.

In response to the TSST, quantity of plasma cfDNA and cf-mtDNA levels doubled, peaking immediately after the end of stress exposure. We addressed the question of whether there might be a stress-specific cfDNA signature by comparing cfDNA increases, fragment length, and DNA methylation as an indicator of cellular origin between psychosocial stress exposure and physical stress in the same individuals. Following strenuous physical exercise, we found a fivefold increase in cfDNA after cessation of exercise, as previously shown by Beiter et al.^6^. Furthermore, fragment analysis of cfDNA revealed a distinct fragmentation pattern of stress-provoked cfDNA levels, with physical strain inducing cfDNA molecules of smaller sizes.

In addition to cfDNA increase and alterations in the number of small fragments, we observed divergent patterns of DNA methylation over time in the CpG island of our reporter gene’s (*HOXA5*) promoter when comparing both stress paradigms (Fig. 1c). There was a shift towards lower cfDNA methylation after psychosocial compared to increased methylation after physical stress. CfDNA which leads to lowered total methylation of cfDNA must derive from cells with a hypomethylated *HOXA5* gene locus, whereas cfDNA that increases total DNA methylation of plasma cfDNA at that specific genomic locus must derive from cells with a hypermethylated *HOXA5* locus. According to various databases (blueprint-epigenome, University of California, Santa Cruz (UCSC) genome browser), the *HOXA5* gene locus is hypomethylated in brain and neuronal cells and shows hypermethylation in blood and muscle cells. The exact cellular origin remains elusive, but these results do point towards different cellular origins of cfDNA when triggered by psychosocial stress compared to physical exercise, suggesting that the release of cfDNA might not be just disposal of damaged DNA but rather a result of a regulated process.

Whereas we could show differences in quantity and quality between the two conditions, our investigation did not address whether differences in cfDNA signatures between conditions might also reflect differences in function. As a comprehensive understanding of molecular processes involved in the release and function of cfDNA is still lacking, we can only speculate about stimulus-specific downstream effects of different cfDNA populations and release mechanisms at this point. Interestingly, active release of DNA, either from nuclear or mitochondrial genomic content, has recently emerged as a remarkable feature of several white blood cell lineages. Moreover, it has been shown that leukocyte-derived DNA may play a crucial role in the regulation of immune responses and thus may serve as another universal type of communication mechanism to shape immunity at multiple levels^25,26^.

Quick release of cfDNA after psychosocial and physical stress as observed might possibly be attributed to vital NETotic processes rather than to slow processes such as apoptosis and necrosis, raising the possibility that stress-induced cfDNA release might be involved in stress-associated immune system regulation^44^. NETs have emerged as an important and highly conserved innate host defense mechanism and, recently, netting neutrophils have also been observed to occur in the blood in response to strenuous exercise^7^. On the other hand, aberrant or unresolved release of NETs has been documented to contribute to the pathogenesis of diverse auto-inflammatory conditions, vascular inflammation, thrombosis, and cancer^45–48^. However, the proportion to which NETs may contribute to increased cfDNA levels in different conditions is currently unknown.

Another possible source of DNA in the circulation after different stress conditions are extracellular vesicles (EVs). Following appropriate stimuli, most cells release EVs which carry RNA, lipids, and DNA^49–53^ at least on their surface^54^. Interestingly, Lutgendorf et al.^55^ recently reported that social support influences the RNA population of exosomes, associated with improved health outcomes in patients with ovarian carcinoma. Further studies are warranted to identify to which extent EVs contribute to cfDNA increases following stress, and in addition, how EV-specific DNA and RNA populations might influence stress-specific aspects of human behavior and health outcomes.

As a limitation, it needs to be noted that only males were investigated, and that the sample size was modest. Furthermore, it cannot be entirely excluded that the stress associated with catheter placement was responsible for cfDNA increases. However, we believe that this is extremely unlikely, as the catheter was inserted 45 min before testing which is common practice when potential effects of blood draw should be avoided. Should venipuncture lead to cfDNA release, most of this elevation would have already metabolized before start of the stress test, as cfDNA has a half-life of about 15 min. Furthermore, cfDNA values 2 min before testing were low and highly similar between conditions, with differences emerging after stimulation.

Human, animal, and cell culture studies suggest that cfDNA can significantly influence the physiological activity of intact living cells (reviewed in ref. ^56^). Although the underlying mechanisms are poorly understood as of yet, it is evident that regular physical exercise provides ample protection from dysregulation of immune homeostasis that compromises the body’s defense systems, as observed in multiple chronic disorders^57–60^. On the other hand, social and psychosocial stressors have a profound negative impact on proper immune system balance and can lead to mood disorders, such as depression^61^. Necessarily, a better understanding of the acute stress response pattern in health and disease and its differential outcomes is inevitable in facing the epidemic increase in lifestyle-associated physical as well as mental illnesses^57–60,62,63^.

In summary, we could show that psychosocial stress exposure as well as physical exercise lead to increased cfDNA release, with stimulus and/or intensity-dependent differences in magnitude, size, and methylation pattern following different challenge protocols. Our findings support the idea of using cfDNA as a biomarker in experimental stress research in addition to hormone levels such as cortisol or catecholamines. Furthermore, cfDNA could possibly be used for diagnosis or monitoring of treatment progression in stress-related mental disorders and for subgrouping of patients with similarities in stress-related pathophysiological processes. However, studies clarifying the functional physiological role of cfDNA are warranted.

## Electronic supplementary material

Supplementary Information_1

Supplementary Information_2_Table

Supplementary Information_2

Supplementary Information_3

Supplementary Information_4

Supplementary Information_5

Supplementary Information_6

Supplementary Information_7

Supplementary Information_8
