# Supplementary material for: Cell-free DNA release under psychosocial and physical stress conditions
Source: Transl Psychiatry. 2018 Oct 29;8:236. doi: 10.1038/s41398-018-0264-x (PMC6206142; doi:10.1038/s41398-018-0264-x)
Supplement: Supplementary file 1 — Supplementary Information_1 [file 41398_2018_264_MOESM1_ESM.docx]

Supplementary Information 1: Primers

| PCR Primers | Sequence in 5->3 | Amplicon size | Annealing temperature | Primer efficiency/R2 | LOQ | LOD |
| --- | --- | --- | --- | --- | --- | --- |
| *BDNF*  (NM_170731) | fw: CAAGGCAGGTTCAAGAGGCT  rv: GCCGAACTTTCTGGTCCTCA  [0.5 µmol/PCR] | 89 bp | 60 °C | E= 98.8%  R2= 0.999 | 80 | 10 |
| spike-in 103 bp fragment | fw: TGTAAAACGACGGCCAGTGA  rv: GCCTTCCTTCAATTCGCCCT  [0.5 µmol/PCR] | 64 bp | 60 °C | E= 100.2%  R2= 1.000 | 16 | 16 |
| spike-in 306 bp fragment | fw: GTCCAGGGTATGCAGACGAC  rv: CGGGTCAGGATCTGCTGTC  [0.5 µmol/PCR] | 89 bp | 60 °C | E= 100.5%  R2= 0.998 | 32 | 8 |
| spike-in 663 bp fragment | fw: TTTCACAAACAGCACCGGAC  rv: CAGACCATTGGCTGCTCTGA  [0.5 µmol/PCR] | 72 bp | 60 °C | E= 89.9%  R2= 0.998 | 64 | 32 |
| spike-in 2,421 bp fragment | fw: GCGCTTCTTGAGTTACCCCT  rv: TCAGAGTGGTCCATGTTGCC  [0.5 µmol/PCR] | 87 bp | 60 °C | E= 99.7%  R2= 0.999 | 32 | 16 |
| cf-mtDNA  (KJ676545.I) | fw: CTATCCGCCATCCCATACATTG  rv: ATCGTGTGAGGGTGGGACT  [0.5 µmol/PCR] | 85 bp | 60 °C | E= 91.8%  R2= 0.999 | N/A | N/A |
| Pyrosequencing Primers | Bisulfite specific sequence in 5->3 | Amplicon size | Annealing temperature | Sequence analyzed  (after bisulfite treatment) | | |
| *HOXA5* (NM_019102.3) | Fw: AGGTTGGTTTTATTATGATTTATGTGTAG  Rv: Bio-ACCCACATCAACAACAAAAAAAAAATT  [0.2 µmol/PCR]  Sequencing primer:  TTGAGTATTTAGGGGTAGATTTG  [0.5 µmol] | 173 bp | 56 °C | 5´GGGTTGGG**CG**GG**CG**G  **CG**C**CG**GGCT**CG**GCT**CG**CT  CTG**CG**CACT**CG**CCTGCT**CG**´3 | | |
