# Supplementary material for: Cell-free DNA release under psychosocial and physical stress conditions
Source: Transl Psychiatry. 2018 Oct 29;8:236. doi: 10.1038/s41398-018-0264-x (PMC6206142; doi:10.1038/s41398-018-0264-x)
Supplement: Supplementary file 2 — Supplementary Information_2_Table [file 41398_2018_264_MOESM2_ESM.docx]

**Supplementary Information 2:**

| **Well** | **Target** | **Sample** | **Cq** | **Copies** | **Mean Copies** | **Desired Copies** | **Yield %** |
| --- | --- | --- | --- | --- | --- | --- | --- |
| I16 | 103 bp ARC | cfDNA | 23,66 | 1374 |  |  |  |
| I17 | 103 bp ARC | cfDNA | 23,88 | 1184 |  |  |  |
| I18 | 103 bp ARC | cfDNA | 24,1 | 1015 | 1191 | 1336 | 89 |
| I19 | 103 bp ARC | Spike In | 19,17 | 28890 |  |  |  |
| I20 | 103 bp ARC | Spike In | 19,26 | 27150 |  |  |  |
| I21 | 103 bp ARC | Spike In | 19,43 | 24110 | 26717 |  |  |
| J16 | 306 bp ARC | cfDNA | 24,09 | 1145 |  |  |  |
| J17 | 306 bp ARC | cfDNA | 24,16 | 1097 |  |  |  |
| J18 | 306 bp ARC | cfDNA | 24,16 | 1094 | 1112 | 1377 | 81 |
| J19 | 306 bp ARC | Spike In | 19,34 | 31710 |  |  |  |
| J20 | 306 bp ARC | Spike In | 19,59 | 26690 |  |  |  |
| J21 | 306 bp ARC | Spike In | 19,73 | 24230 | 27543 |  |  |
| K16 | 2421 bp ARC | cfDNA | 23,23 | 1542 |  |  |  |
| K17 | 2421 bp ARC | cfDNA | 23,39 | 1372 |  |  |  |
| K18 | 2421 bp ARC | cfDNA | 23,68 | 1123 | 1346 | 1427 | 94 |
| K19 | 2421 bp ARC | Spike In | 18,92 | 30340 |  |  |  |
| K20 | 2421 bp ARC | Spike In | 19,03 | 28260 |  |  |  |
| K21 | 2421 bp ARC | Spike In | 19,09 | 26990 | 28530 |  |  |
