# Supplementary figures and images for: Cell-free DNA release under psychosocial and physical stress conditions
Source: Transl Psychiatry. 2018 Oct 29;8:236. doi: 10.1038/s41398-018-0264-x (PMC6206142; doi:10.1038/s41398-018-0264-x)

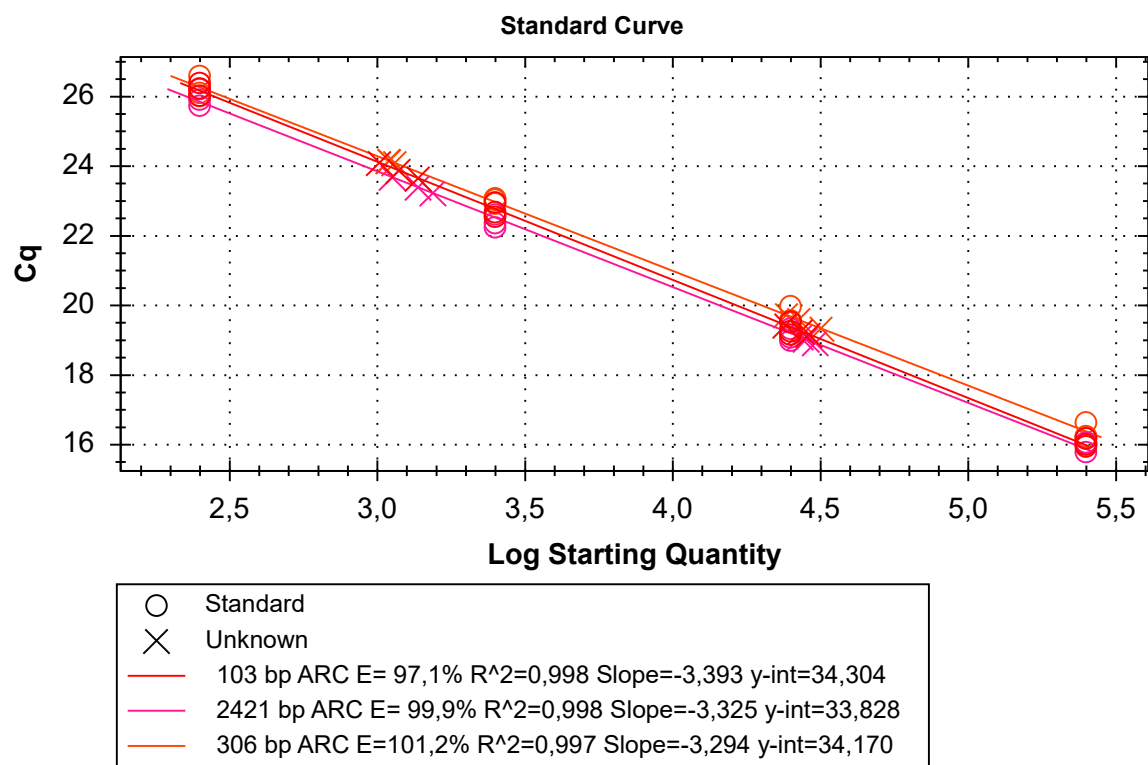

Supplement: Supplementary file 3 — Supplementary Information_2 [file 41398_2018_264_MOESM3_ESM.pdf]

### Fragment sizes psychosocial stress

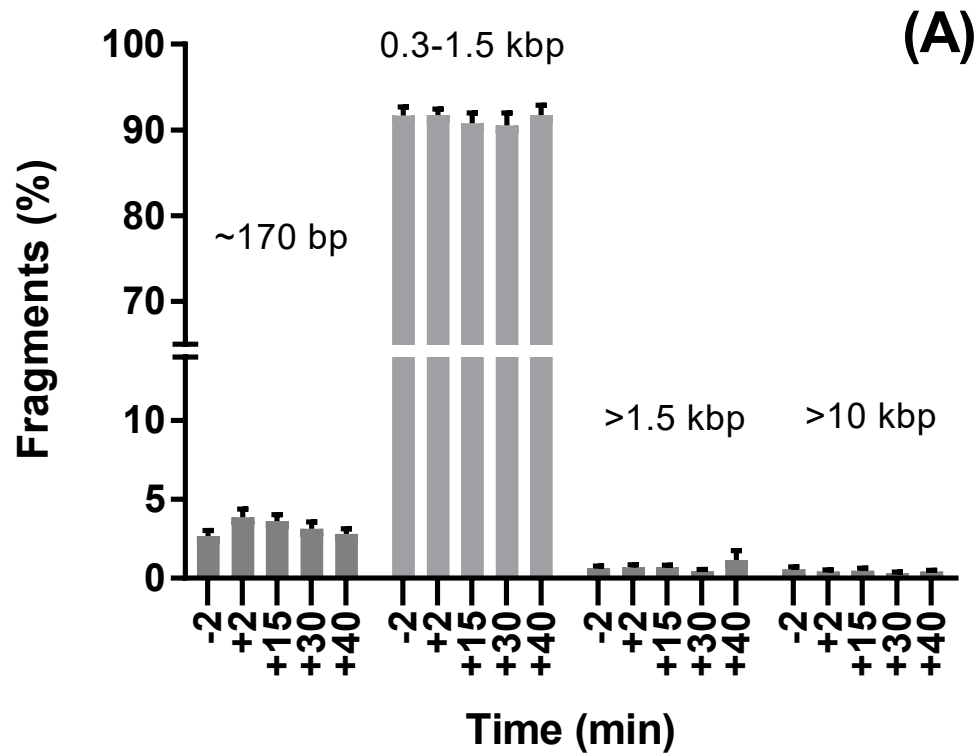

### Fragment sizes physical stress

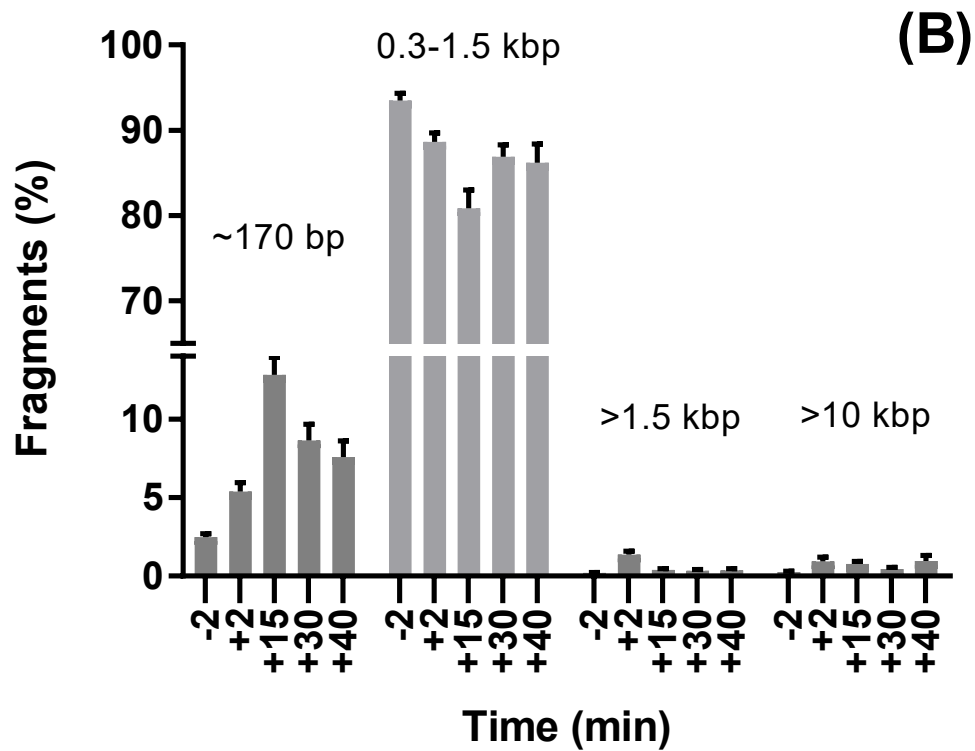

Supplement: Supplementary file 4 — Supplementary Information_3 [file 41398_2018_264_MOESM4_ESM.pdf]

# Alpha-Amylase psychosocial vs. physical stress

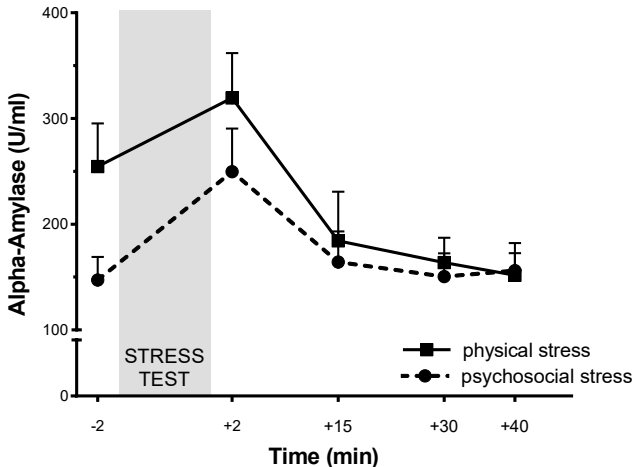

Supplement: Supplementary file 5 — Supplementary Information_4 [file 41398_2018_264_MOESM5_ESM.pdf]

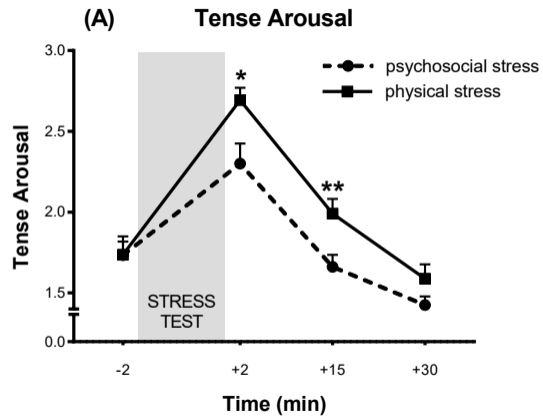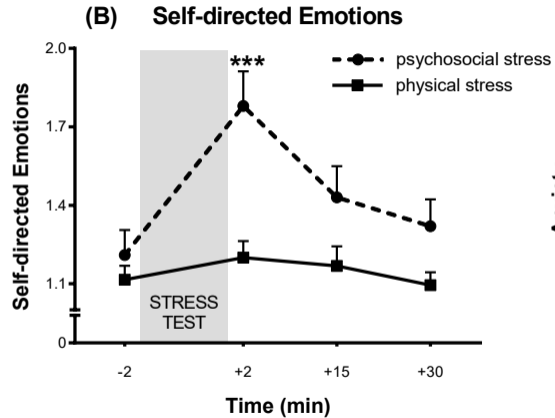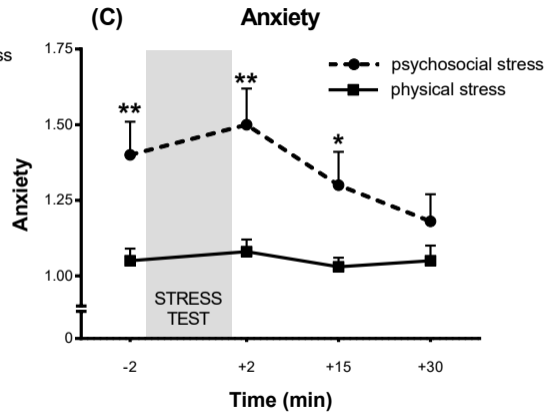

Supplement: Supplementary file 6 — Supplementary Information_5 [file 41398_2018_264_MOESM6_ESM.pdf]

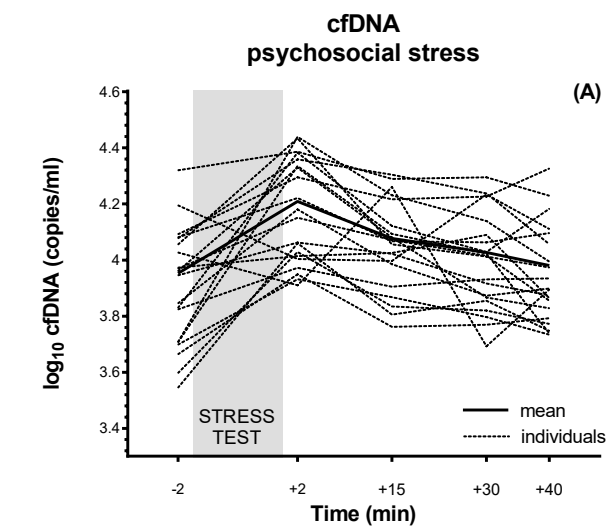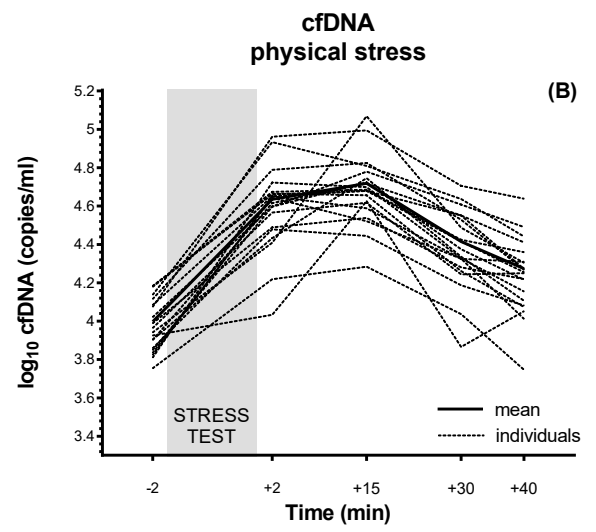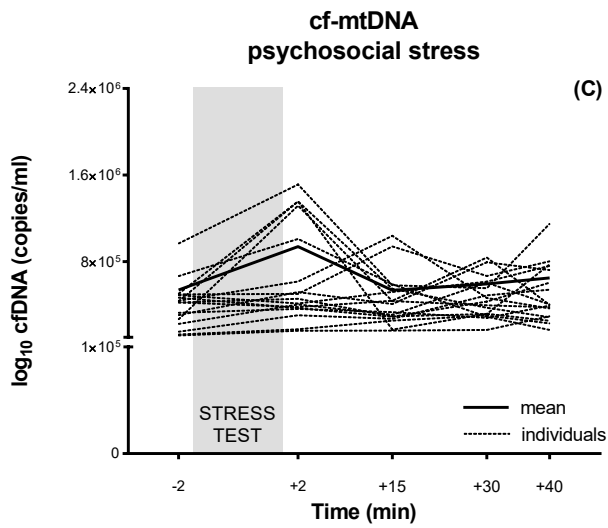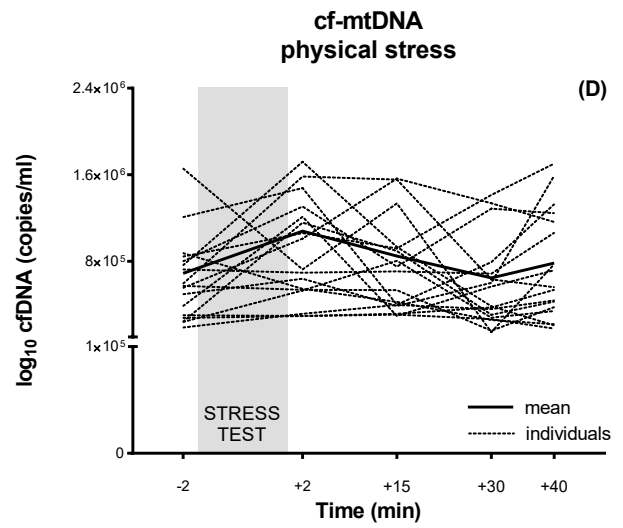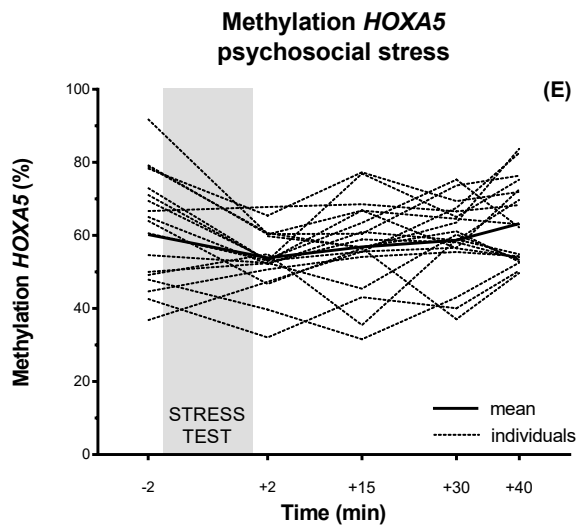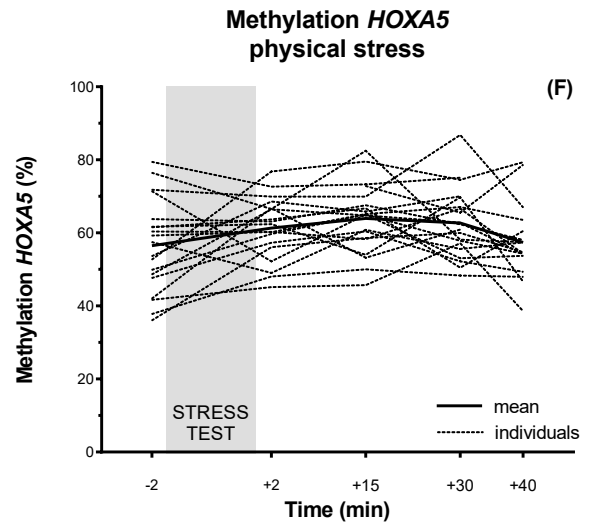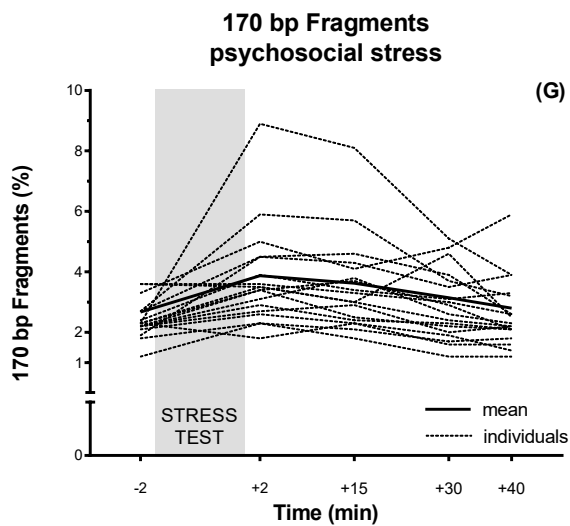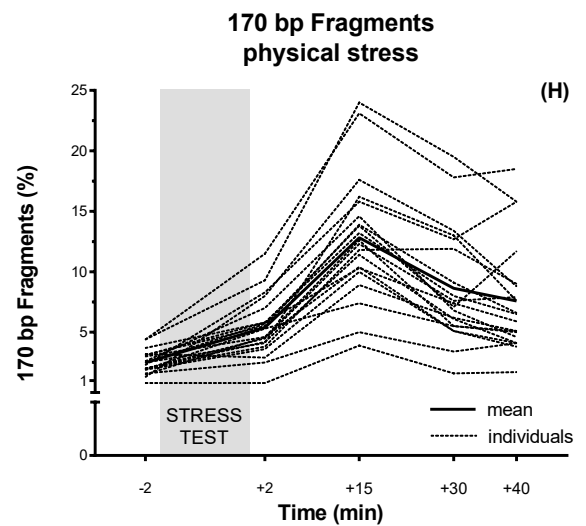

Supplement: Supplementary file 7 — Supplementary Information_6 [file 41398_2018_264_MOESM7_ESM.pdf]

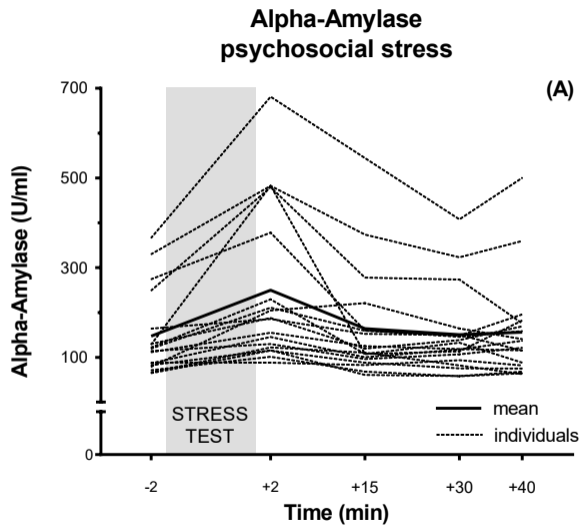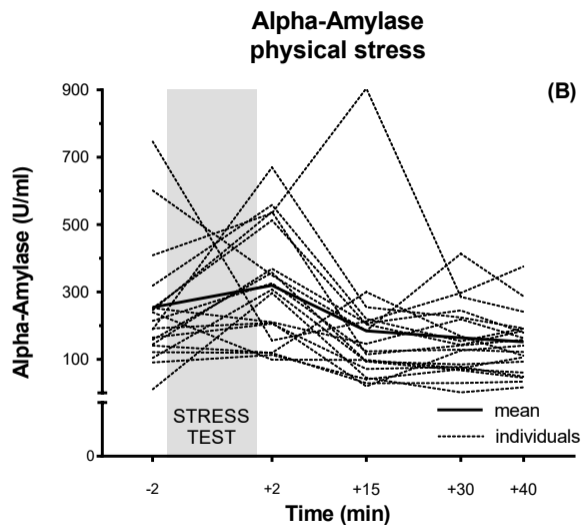

Supplement: Supplementary file 9 — Supplementary Information_8 [file 41398_2018_264_MOESM9_ESM.pdf]
