# Supplementary material for: Cell-free DNA release under psychosocial and physical stress conditions
Source: Transl Psychiatry. 2018 Oct 29;8:236. doi: 10.1038/s41398-018-0264-x (PMC6206142; doi:10.1038/s41398-018-0264-x)
Supplement: Supplementary file 8 — Supplementary Information_7 [file 41398_2018_264_MOESM8_ESM.pdf]

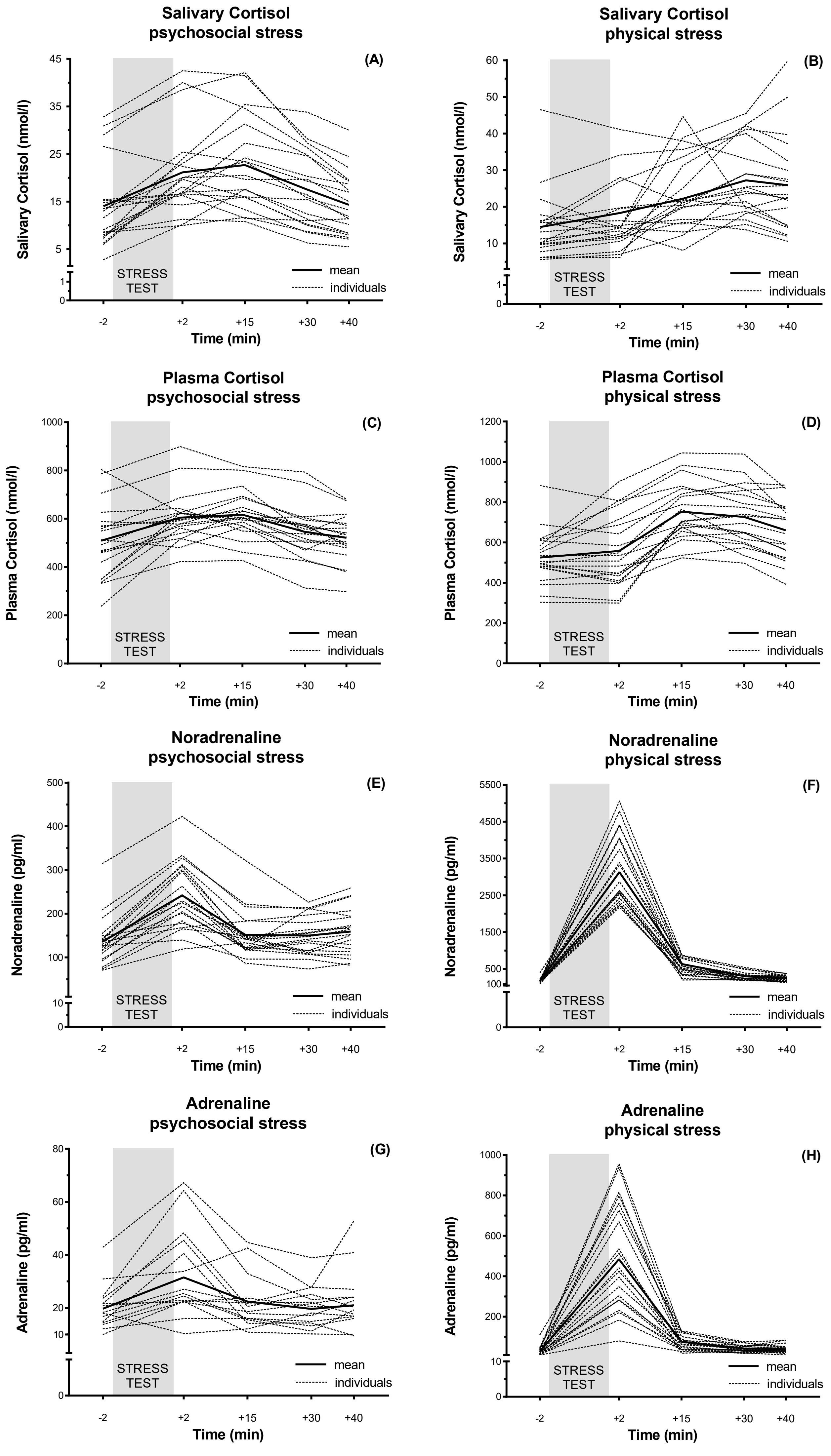

Salivary Cortisol psychosocial stress
(A)
Salivary Cortisol (nmol/l)
45
35
25
15
5
1
0
STRESS TEST
mean
individuals
-2
+2
+15
+30
+40
Time (min)
Salivary Cortisol physical stress
(B)
Salivary Cortisol (nmol/l)
60
50
40
30
20
10
1
0
STRESS TEST
mean
individuals
-2
+2
+15
+30
+40
Time (min)
Plasma Cortisol psychosocial stress
(C)
Plasma Cortisol (nmol/l)
1000
800
600
400
200
0
STRESS TEST
mean
individuals
-2
+2
+15
+30
+40
Time (min)
Plasma Cortisol physical stress
(D)
Plasma Cortisol (nmol/l)
1200
1000
800
600
400
200
0
STRESS TEST
mean
individuals
-2
+2
+15
+30
+40
Time (min)
Noradrenaline psychosocial stress
(E)
Noradrenaline (pg/ml)
500
400
300
200
100
10
0
STRESS TEST
mean
individuals
-2
+2
+15
+30
+40
Time (min)
Noradrenaline physical stress
(F)
Noradrenaline (pg/ml)
5500
4500
3500
2500
1500
500
100
0
STRESS TEST
mean
individuals
-2
+2
+15
+30
+40
Time (min)
Adrenaline psychosocial stress
(G)
Adrenaline (pg/ml)
80
60
40
20
10
0
STRESS TEST
mean
individuals
-2
+2
+15
+30
+40
Time (min)
Adrenaline physical stress
(H)
Adrenaline (pg/ml)
1000
800
600
400
200
10
0
STRESS TEST
mean
individuals
-2
+2
+15
+30
+40
Time (min)
